# Postoperative CYFRA 21-1 and CEA as prognostic factors in patients with stage I pulmonary adenocarcinoma

**DOI:** 10.18632/oncotarget.17611

**Published:** 2017-05-04

**Authors:** Ying He, Yong Cui, Dong Chang, Tianyou Wang

**Affiliations:** ^1^ Department of Thoracic Surgery, Beijing Friendship Hospital, Capital Medical University, Beijing, China

**Keywords:** CYFRA21-1, CEA, pulmonary adenocarcinoma, surgical resection, postoperative prognosis

## Abstract

**Background:**

Patients with pathological stage I pulmonary adenocarcinoma have different postoperative prognosis. The aim of this study is to evaluate the prognostic significance of preoperative and postoperative serum levels of carcinoembryonic antigen (CEA) and CYFRA 21-1 in patients with pathological stage I pulmonary adenocarcinoma.

**Material and Methods:**

We retrospectively reviewed the data of 123 patients who had undergone a complete resection for pathological stage I pulmonary adenocarcinoma between 2004 and 2014. The clinical data of each patient including age, gender, preoperative and postoperative serum CEA and CYFRA 21-1 levels, and pathologic stage, was collected for analysis.

**Results:**

The CYFRA 21-1 and CEA level was persistently normal in 80.5% and 77.2% of all patients with p-stage I ADC, respectively. The preoperative level was elevated and postoperative level declined to normal for CYFRA 21-1 and CEA were in 10.6% and 13.0% of all patients, respectively. The postoperative CYFRA 21-1 and CEA level were high in 8.9% and 9.8% of all patients, respectively. The postoperative 5-year survival rate of patients with normal, only preoperative high, and postoperative high CYFRA 21-1 level was 92.6%, 92.3% and 43.8%, respectively. There was a significant difference between postoperative high group and the other two groups (*p* = 0.002). The postoperative 5-year survival rate of patients with normal, only preoperative high, and postoperative high CEA level was 90.8%, 92.3%, and 70.1%, respectively. There was a significant difference between postoperative high group and the other two groups (*p* = 0.019). In univariate analysis, degree of differentiation, visceral pleural invasion, tumor size, and pTNM stage, was found to be significant independent prognostic factor (*p* = 0.014). Multivariate analysis showed that pTNM stage, postoperative CYFRA 21-1 high level, and postoperative CEA high level was related to the poor prognosis.

**Conclusions:**

Patients of p-stage I ADC with postoperative high serum level of either CEA or CYFRA 21-1 had poor prognosis. Carefully followed-up might be necessary to rule out occult metastasis for these patients, and further clinical studies will be necessary to evaluate the efficacy of adjuvant chemotherapy or target therapy. Postoperative high serum level of CEA or CYFRA 21-1 might be a subtype of p-stage I ADC.

## INTRODUCTION

Lung cancer is among the most prevalent and lethal cancers worldwide [1], and non-small cell lung cancer (NSCLC) comprises approximately 85% of lung cancer cases [2]. The 5-year survival rate of patients with pathological stage I NSCLC was only 60-85% [3–4]. The wide range of survival rates suggests that patients who undergo surgery are a heterogeneous population with different disease progression. Adenocarcinoma (ADC) has a relatively higher possibility of developing distant micrometastasis without local progression in patients with early TNM stage. A Japanese randomized Phase III trial of adjuvant chemotherapy for completely resected pathological stage I NSCLC showed a survival benefit for patients with ADC; however, there was no benefit for patients with squamous carcinoma. [5]. For these patients the tumor cells might have spread to lymph nodes or other tissues, and currently available clinical practices have failed to detect these micrometastasis. Therefore, tumor markers might be promising in selecting the optimal treatment modality.

Cytokeratin 19 fragments (CYFRA 21-1) and carcinoembryonic antigen (CEA) are popular serum tumor markers used for NSCLC. The serum CYFRA 21-1 level has been shown to be a sensitive tumor marker in the squamous cell type [6]. However, the serum CEA level has more precise diagnostic values in ADC than other cell type of NSCLC. While the information is limited on the study of CEA and CYFRA21-1 for prognosis by simultaneously considering their preoperative and postoperative levels [7–10]. This study retrospectively investigated the clinical significance of the perioperative serum level of CYFRA 21-1 and CEA as prognostic factors in patients with pathological stage I ADC.

## PATIENTS AND METHODS

The hospital records of 123 patients who underwent a complete resection for pathological stage I pulmonary ADC at the Beijing Friendship Hospital in China between 2004 and 2015 were reviewed. The serum levels of CYFRA 21-1 and CEA were examined as a part of the routine preoperative evaluation within 30 days prior to surgery, and within three months after surgery when they accept routine clinical examination. The CYFRA21-1 levels were analyzed by electrochemiluminescence method by using Roche Elecsys (Roche Diagnostics Limited, Shanghai, China). The serum CEA levels was measured by chemiluminesent immune-assay method, and the reagent kit was manufactured by Abbott (Abbott Park, Illinois, USA). The normal upper limit was set as 3.3 ng/mL and 5.0 ng/mL for CYFRA21-1 and CEA, respectively. The other preoperative assessments included roentgenography and computed tomography (CT) of the chest and upper abdomen, bone scintigraphy, and magnetic resonance imaging (MRI) of the brain. Ten patients had lung cancer recurrence or metastasis that was confirmed by CT, MRI, bone scintigraphy, and PET-CT. The patients’ medical records were carefully reviewed including age, gender, smoking history, past medical history, degree of differentiation, tumor size, tumor location, visceral pleural invasion, pTNM, preoperative and postoperative serum CEA and CYFRA21-1 levels, and survival situation (Table 1). The histopathological findings were classified according to the World Health Organization criteria, and the UICC TNM staging system (7th edition) was employed [11–12]. Follow-up information was obtained from all patients through office visits or telephone interviews either with the patient or with a relative. The patients were evaluated every 3 months by chest roentgenography, and chest CT scans and bone scintigraphy were performed every 6 months for the first 2 years after surgery and annually thereafter.

**Table 1:** Clinicopathological factors of the 123 patients with p-stage I ADC

Factors		CYFRA21-1				CEA			
	Total	NN group	HN group	HH/NH group	*p* value^a^	NN group	HN group	HH/NH group	*p* value^a^
	*n*=123	*n*=99	*n*=13	*n*=11		*n*=95	*n*=16	*n*=12	
Age					0.149				0.736
<65y	73	59	10	4		58	9	6	
≧65y	50	40	3	7		37	7	6	
Gender					0.513				0.538
Male	47	39	3	5		34	8	5	
Female	76	60	10	6		61	8	7	
Smoking history					0.365				0.01
No	92	71	11	10		76	7	9	
Yes	31	28	2	1		19	9	3	
Past medical history					0.325				0.380
No	48	41	5	2		38	4	6	
Yes	75	58	8	9		57	12	6	
Differentiation					0.567				0.122
Well	28	25	2	1		25	0	3	
Moderate	82	65	9	8		60	14	8	
Poor	13	9	2	2		10	2	1	
VPI					0.56				0.262
Absent	65	57	3	5		54	6	5	
Present	58	42	10	6		41	10	7	
Tumor size(mm)					0.082				0.005
0-20	71	62	5	4		62	4	5	
21-50	52	37	8	7		33	12	7	
PTNM					0.127				0.452
Ia	61	53	3	5		50	6	5	
Ib	62	46	10	6		45	10	7	

Note:

CEA: carcinoembryonic antigen;

CYFRA21-1: the soluble fragment of cytokeratins-19;

VPI: visceral pleural invasion;

NN: continuously normal;

HN: preoperative high level, declined to normal level after operation;

HH/NH: postoperative high level;

CYFRA21-1, CEA, and other clinical variables were compared by using Fisher's exact test or chi-square test. The OS curves were plotted by using Kaplan Meier method in the survival analysis, and the significant difference was tested by Log-rank method. Prognostic factor were identified by univariate Cox proportional hazards model. The independent risk factors for prognosis were determined by multivariate Cox proportional hazards model after incorporating the results of univariate analysis into each group. The software used in the study was SPSS20.0 (IBM, Armonk, New York, USA). The significant difference was judged if the p-value was less than 0.05.

## RESULTS

These were 135 patients who had undergone a complete resection for p-stage I ADC. By August 20, 2015, 123 patients received follow-ups and 12 patients lost communications. The 123 patients included 47 males and 76 females. The mean age of the patients was 67.3 years (range:29-85). The median follow-up time was 46.2 months with the range of 5-131 months. Among 123 patients, 107 patients were alive, 16 passed away caused by original diseases.

The CYFRA 21-1 and CEA level were persistently normal in 77.2% and 80.5% of all patients with p-stage I ADC, respectively. The preoperative CYFRA 21-1 and CEA level were high but declined to normal after surgery in 10.6% and 13.0% of all patients, respectively. Both of the CYFRA 21-1 and CEA preoperative high level were significantly correlated with tumor size (*p* = 0.005 and 0.011, respectively). The postoperative CYFRA 21-1 and CEA level were high in 8.9% and 9.8% of all patients, respectively. There was 2 patients whose preoperative CYFRA 21-1 levels were normal but postoperative levels were high, and two patients whose preoperative CEA level was normal but postoperative level was high.

The postoperative 5-year survival rate of patients with normal, only preoperative high, and postoperative high CYFRA 21-1 level was 92.6%, 92.3% and 43.8%, respectively (There are 8 patients received their surgery after 2013, so we use the postoperative survival curve to calculate the postoperative 5-year survival rate). There was a significant difference between postoperative CYFRA 21-1 high level group and other two groups (*p* = 0.002). The postoperative 5-year survival rate of patients with normal, only preoperative high, and postoperative high CEA level was 90.8%, 92.3% and 70.1%, respectively. There was a significant difference between postoperative high CEA level group and the other two groups (*p* = 0.019), and that is similar to CYFRA 21-1.

In univariate analysis, degree of differentiation (95% confidence interval 1.286-9.217, *p* = 0.014), visceral pleural invasion (95% confidence interval 0.803-8.477, *p* = 0.111), tumor size (95% confidence interval 2.139-43.756, *p* = 0.006), pTNM stage (95% confidence interval 0.130-1.372, *p* = 0.152), postoperative high CYFRA 21-1 level (95% confidence interval 1.432-5.150, *p* = 0.002), and postoperative high CEA level (95% confidence interval 1.092-4.155, *p* = 0.026) was found to be significant independent prognostic factor.

**Figure 1:**
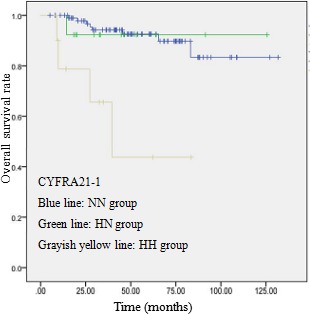
OS curve of 123 patients with p-stage I ADC based on serum CYFRA21-1 levels A significant difference in OS was observed between HH group and the others (*P* = 0.005, log-rank test)

No significant differences were observed between the patients with/without preoperative high level of CYFRA 21-1 or CEA. In a multivariate analysis using these variables (age, gender, tumor size, and CEA and CYFRA 21-1 levels), the tumor size (>2cm), postoperative CYFRA 21-1 high level, and postoperative CEA high level, was found to be significant independent prognostic factor for OS. The higher preoperative CYFRA 21-1 or CEA level was not found to be a significant independent prognostic factor, if it decline to normal level after operation. (Table 3).

**Table 2:** Predictors of OS according to univariate analysis

Risk factors	OS	
	HR (95% CI)	*p*-value
Age	1.033 (0.975-1.095)	0.268
Gender	1.338 (0.449-3.988)	0.601
Smoking history	1.360 (0.373-4.962)	0.641
Past medical history	0.833(0.270-2.577)	0.752
Differentiation degree	3.443 (1.286-9.217)	0.014
VPI	2.610(0.803-8.477)	0.111
Tumor size (mm)	9.675(2.139-43.756)	0.003
Location	1.412(0.434-4.594)	0.567
pTNM	0.422(0.130-1.372)	0.152
CYFRA21-1 Groups	2.716(1.432-5.150)	0.002
CEA Groups	2.130(1.092-4.155)	0.026

Note:

^a^The log-rank test.

CEA: carcinoembryonic antigen;

CYFRA21-1: the soluble fragment of cytokeratins-19;

VPI: visceral pleural invasion;

NN: continuously normal;

HN: preoperative high level, declined to normal level after operation;

HH/NH: postoperative high level;

CI: confidence interval.

**Table 3:** Predictors of OS according to multivariate analysis

Risk factors	OS		
	HR	95% CI	*p*-value
Tumor size (mm)			
0-20	1	Reference	
21-50	20.855	3.283-132.468	0.001
CYFRA21-1			
NN group	1	Reference	
HN group	0.709	0.080-6.273	0.758
HH group	9.104	1.966-42.163	0.005
CEA			
NN group	1	Reference	
HN group	0.131	0.014-1.231	0.075
HH group	9.599	2.294-40.162	0.002

Note:

CEA: carcinoembryonic antigen;

CYFRA21-1: the soluble fragment of cytokeratins-19;

NN: continuously normal;

HN: preoperative high level, declined to normal level after operation;

HH/NH: postoperative high level;

CI: confidence interval.

Only statistically significant (*p* < 0.05) values were shown.

Clinical characteristics were compared using the chi-square test. Each group's overall survival (OS) was analyzed by using the Kaplan-Meier method. The prognostic factor was evaluated by using the Cox proportional hazards model.

## DISCUSSION

Therefore, tumor markers, which were widely regarded as diagnostic tools, and were commonly used as the indicators for predicting the treatment efficacy of patients with for identifying malignant tumors [7, 8, 13, 14], are required to help to select the optimal treatment modality for individual patients with ADC. The prognostic significance of preoperative CYFRA 21-1 and CEA has been investigated in patients with stage I NSCLC [18–19]. However, the prognostic values of these tumor markers were still controversial. Moreover, most of the studies included not only the ADC, but also the squamous cell carcinoma. The present study focused on the perioperative levels of CYFRA 21-1 and CEA as an indicator of prognosis in patients with stage I ADC who underwent complete resection.

It was reported that the serum level of CYFRA 21-1 in ADC is significantly lower than that in squamous cell carcinoma, the sensitivities of CYFRA 21-1 for ADC, large cell carcinoma, and squamous cell carcinoma were 39%, 36%, and 62%, respectively [13, 16]. CEA is mainly associated with ADC, elevated levels of serum CEA have been reported in 35-60% of NSCLC patients [15–17]. In this study, CEA level was high in 13.0% before surgery and in 9.8% after surgery of all patients with stage I ADC. Similar to CEA, CYFRA21-1 level was high in 10.6% before surgery and in 8.9% after surgery. Meanwhile, this study demonstrated that CYFRA21-1 and CEA high level before operation was significantly correlated with tumor size (*p* = 0.005 and 0.011, respectively).

**Figure 2:**
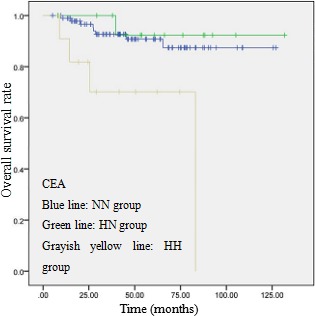
OS curve of 123 patients with p-stage I ADC based on serum CEA levels A significant difference in OS was observed between HH group and the others (*P* = 0.005, log-rank test).

The prognostic values of these tumor markers were still controversial. Matsuoka et al. reported that preoperative CEA was a good candidate that could be used to select the high-risk group, but CYFRA 21-1 was not associated with cancer recurrence postoperatively [18]. However, Hanagiri et al. reported that preoperative CYFRA 21-1, not CEA, was a significant independent prognostic factor for patients with stage I NSCLC [20]. Mizuguchi et al. demonstrated that elevated preoperative CYFRA 21-1 and CEA were both unfavorable prognostic factors by univariate analysis, however, while CYFRA 21-1 was an independent prognostic factor in patients with stage I NSCLC, CEA was not [21]. In contrast, Blankenburg et al. indicated that preoperative elevation of CYFRA 21-1 and CEA was not associated with unfavorable survival [19]. The present study showed that high CYFRA 21-1 or CEA serum levels after surgery indicate poor prognosis in patients with stage I ADC. That is consistent with the conclusion of Kozu [9] and Wang [6], both of them reported that postoperative serum CEA levels had prognostic significance for patients with completely resected pathological-stage I non-small cell lung cancer. While, preoperative high level of CYFRA 21-1 was not associated with poor prognosis in patients with stage I ADC, if it declined to normal after surgery. That is consistent with Blankenburg's study. [19]

Our results highlight the prognostic value of postoperative serum levels either CYFRA 21-1 or CEA for patients with stage I ADC. This parameter might be considered as an independent factor affecting survival beyond the current TNM staging system for stage I ADC. In conclusion, In conclusion, high postoperative serum CYFRA 21-1 or CEA level was risk factors for poor survival in p-stage I ADC patients. Carefully followed-up might be necessary to rule out occult metastasis for these patients, and further clinical studies will be necessary to evaluate the efficacy of adjuvant chemotherapy or target therapy.
